# Hereditary Multiple Gastrointestinal Atresia associated with Choledochal Cyst: A Rare Entity with Management Dilemma 

**Published:** 2014-07-10

**Authors:** Raj P, Sinha SK, Ramji S, Sarin YK

**Affiliations:** Department of Pediatric Surgery, Maulana Azad Medical College and associated Lok Nayak Hospital, New Delhi; 1 Department of Neonatology, Maulana Azad Medical College and associated Lok Nayak Hospital, New Delhi

**Keywords:** Multiple intestinal atresia, Choledochal cyst, Polyhydramnios

## Abstract

Multiple intestinal atresias are rare and its treatment is challenging. Here, we present a case of multiple gastro-intestinal atresia associated with choledochal cyst posing us a surgical challenge.

## CASE REPORT

A full-term, normally vaginal delivered, 10-hour-old male baby, born out of consanguineous marriage, presented with bilious vomiting and mild upper abdominal fullness. Antenatal USG had shown gross polyhydramnios and dilated bowel loops. A 3-year-old sibling is known case of classical galactosemia. Abdominal examination revealed epigastric fullness. Abdominal roentgenograph showed single dilated gastric bubble with no distal gas and few calcifications in lower abdomen (Fig. 1). Exploratory laparotomy revealed multiple atresias involving duodenum, jejunum, ileum and colon. There were intra-luminal calcifications between the atretic segments. The stomach and duodenum were grossly dilated. Most of the jejunum and ileum were atretic with no lumen and inter atretic segment filled with calcified putty-like material. The large gut from caecum to transverse colon was filled with calcified material. There were further atresia present in the descending and sigmoid colon. Additionally, there was a large choledochal cyst measuring 5x4 cms (Fig. 2). We initially planned for multiple resection and anastomoses, but in view of the long atretic segments with no lumen, calcified intra-luminal contents and intra-operative multiple bronchospasms, only resection of proximal jejunum was done. End duodenostomy and jejunostomy were done. Rest of the jejunum, ileum and large bowel till transverse colon were left as such and descending colostomy was done. Tube-drainage of choledochal cyst was done (Fig. 3). A central line for total parenteral nutrition was also placed in internal jugular vein. Post-operatively, the baby was kept on total parenteral nutrition (TPN) for three weeks. The baby had persistent gastric distention and repeat x-ray showed a single bubble (missed pyloric atresia). On day 24, the baby expired due to TPN induced nosocomial sepsis, with blood culture positive for acinetobacter. 

**Figure:**
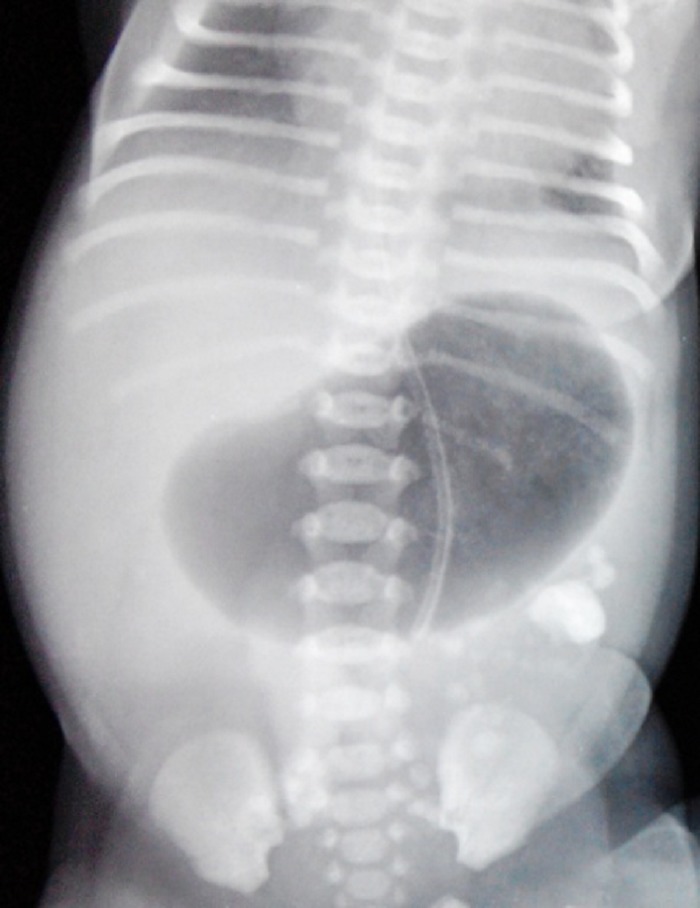
Figure 1: Abdominal radiograph showing single gastric bubble and calcification in left iliac fossa.

**Figure:**
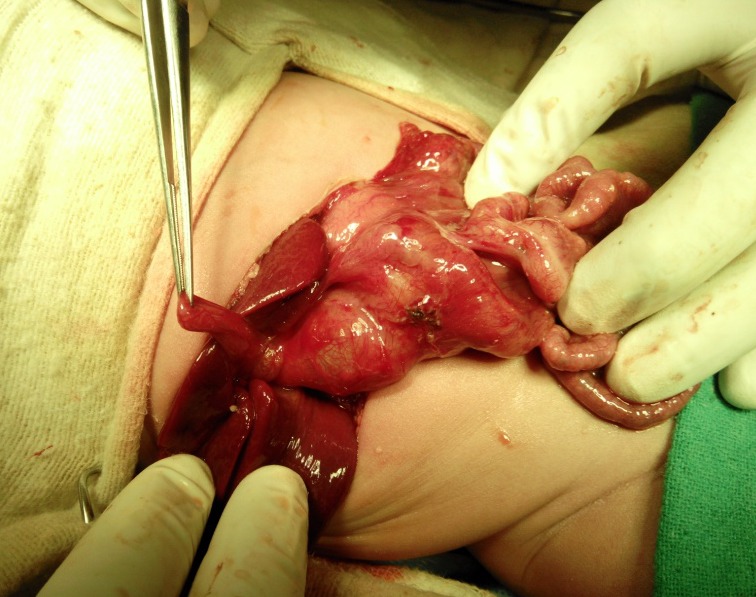
Figure 2: Gall bladder with choledochal cyst. Also note dilated duodenum and atretic small bowel with multiple atresia.

**Figure:**
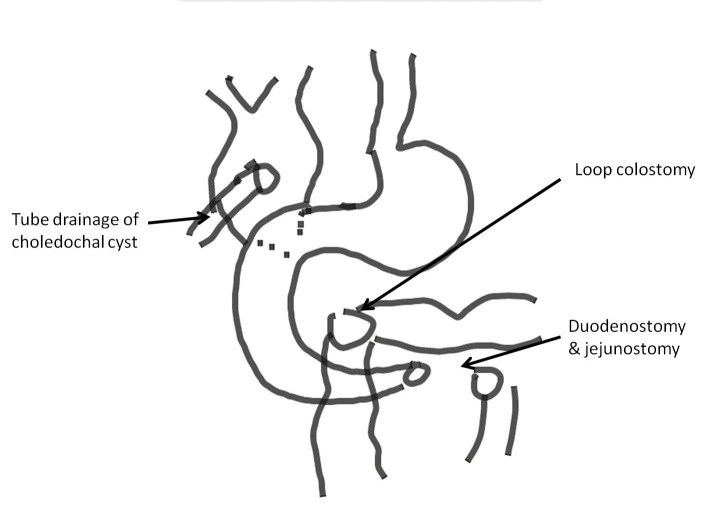
Figure 3: Surgical procedure: external drainage of choledochal cyst with duodenostomy and jejunostomy and loop colostomy.

## DISCUSSION

Multiple intestinal atresia (MIA) is different from duodenal and jejunal atresia because of the extent of involvement from stomach to anus; and present a unique constellation of clinical, radiologic and pathologic findings secondary to malformative process occurring during early intrauterine life.[1] It has been seen in children with common ancestry.[2] Severe combined immunodeficiency is also associated.[3] Transmission pattern in several reported families with MIA had been known to be consistent with autosomal recessive inheritance. TTC7A was the likely causal gene for MIA.[4] Associated anomalies include malrotation, cystic dilatation of bile duct, omphalocele, cardiac anomalies, and congenital cystic adenomatoid malformation.[5] The index case had associated choledochal cyst. We hypothesize that in view of both proximal and distal obstruction of duodenum, the back-pressure led to cystic dilation of common bile duct and thus formation of choledochal cyst.

MIA poses a serious surgical challenge. Surgical treatment has included tapering enteroplasty and resection and primary anastomoses.[6] Standard surgical therapy may not allow maximal preservation of residual bowel because of trimming process required for a safe and secure anastomosis. In addition, multiple anastomoses may require excessive operating time, jeopardizing the newborn’s clinical status. Hatch and Schaller described a “shish-kebab” technique in 1986, to perforate multiple membranous obstructions as an alternative to multiple resections.[7] Use of intraluminal Silastic stents to support multiple hand-sewn anastomoses is also described in literature.[8] However, none of them are feasible in these neonates in which whole of intestine is atretic with no intervening segment of normal intestine. Major cause of morbidity and mortality is short gut syndrome. There are no survivals reported in literature for the neonates with MIA. [5]

## Footnotes

**Source of Support:** Nil

**Conflict of Interest:** The author is editor of the journal but the manuscript is handled independently by other editors and he is not involved in decision making of the manuscript.

